# Experience and perception of utilizing virtual clinic in neurological assessment in Saudi Arabia

**DOI:** 10.3389/fneur.2023.1111254

**Published:** 2023-02-17

**Authors:** Mohammed Hmoud, Hassan K. Salamatullah, Dania E. Faidah, Seraj Makkawi

**Affiliations:** ^1^Department of Medicine, Ministry of the National Guard-Health Affairs, Jeddah, Saudi Arabia; ^2^King Abdullah International Medical Research Center, Jeddah, Saudi Arabia; ^3^College of Medicine, University of Bisha, Bisha, Saudi Arabia; ^4^College of Medicine, King Saud Bin Abdulaziz University for Health Sciences, Jeddah, Saudi Arabia

**Keywords:** virtual clinic, neurology, tele-neurology, neurologists, neurological assessment, Saudi Arabia

## Abstract

**Introduction:**

The World Health Organization defined electronic health as “the unified usage of information technology and electronic communications in the health sector.” In the Kingdom of Saudi Arabia, outpatient encounters were largely shifted to virtual clinics due to the crisis caused by COVID-19. This study aimed to evaluate the neurology consultants', specialists', and residents' experience and perception of utilizing virtual services for neurological assessment in Saudi Arabia.

**Methods:**

This cross-sectional study was conducted by sending an anonymous online survey to neurologists and neurology residents in Saudi Arabia. The survey was developed by the authors and contained three main sections: demographics, subspecialty and years of experience after residency, and virtual clinics during the coronavirus disease 2019 (COVID-19) pandemic.

**Result:**

A total of 108 neurology-practicing physicians in Saudi Arabia responded to the survey. Overall, 75% experienced virtual clinics, and 61% of them used phones for consultation. In neurology clinical practice, there was a significant difference (*P* < 0.001) regarding the teleconsultations for follow-up patients compared to the newly referred patients, being more suitable for the follow-up cases. Additionally, most neurology practicing physicians showed more confidence in performing history-taking tasks virtually (82.4%) than in physical examination. However, it was found that consultants were significantly (*P* < 0.03) more confident to virtually perform the cranial nerve, motor, coordination, and extrapyramidal assessments than the neurology residents. Physicians deemed it more suitable to conduct teleconsultations for patients with headaches and epilepsy than for those with neuromuscular and demyelinating diseases/multiple sclerosis. Furthermore, they agreed that patients' experiences (55.6%) and physicians' acceptance (55.6%) were the two main limitations to implementing virtual clinics.

**Discussion:**

This study revealed that neurologists were more confident in performing history-taking in virtual clinics than in physical exams. On the contrary, consultants were more confident in handling the physical examination virtually than the neurology residents. Moreover, the most accepted clinics to be handled electronically were the headache and epilepsy clinics in comparison to the other subspecialties, being mainly diagnosed using history. Further studies with larger sample sizes are warranted to observe the level of confidence in performing different duties in neurology virtual clinics.

## 1. Introduction

The World Health Organization defines electronic health (e-health) as “the unified usage of information technology and electronic communications in the health sector.” The Ministry of Health proceeded to target tangible progress in e-health in 2011 by announcing the e-health vision: “safe, efficient health system, based on patient-centered care, standard-oriented, and supported by the e-health (1).” The words “e-health” and “telemedicine” are often used interchangeably in the literature. However, “e-health” is a broader term that includes clinical and non-clinical aspects of health, such as education and training. Notably, the term “telemedicine” was used as early as 1940, but it did not start to rapidly grow until recently (2).

In the Kingdom of Saudi Arabia, outpatient encounters were largely shifted to virtual clinics due to the crisis caused by the coronavirus disease 2019 (COVID-19) pandemic. With the initial cases identified in December 2019 and later declared as a global pandemic in March 2020, arose the need for social distancing, the use of personal protective equipment, the closure of big economies partially or completely, and the adjustment in patient clinical encounters and evaluations (3).

Globally, the use of telemedicine has been progressing ever since. For instance, in neurology clinics, telemedicine was largely adopted in the management of patients with dementia, as well as in the prevention and acute management of stroke. However, until the use of virtual clinics was mandated due to the current situation of the pandemic, the progress was slow. From an advantageous perspective, telemedicine can offer information exchange and medical services to overcome time constraints, different geographical locations, and social and cultural barriers (3).

On the other hand, multiple barriers and obstacles to telemedicine were examined in the literature. Many attempts to apply virtual clinics and telemedicine found to have difficulties related to acceptance by the physician and the patient, infrastructure (including internet, software, and needed devices), and shortage of funding. In order to overcome different obstacles, different health-related departments in the United States managed to loosen their restrictive policies (2–4).

In neurology, huge progress was noticed regarding telestroke utilization. Its concept was initially coined in 1999 by Levin and Goman concerning the care of patients with stroke using telephone lines and video conferencing. Currently, this service extends to rural and underserved areas to provide an expert level of care. This step provided more eligible patients with tissue plasminogen activators, who had outcome similar to patients treated in person (2, 5, 6). Furthermore, with regard to neuromuscular diseases such as amyotrophic lateral sclerosis (ALS), there were some developed methods to monitor the patients remotely. For example, ALS functional rating scale revised (ALSFRSr) can be self-administrated remotely to monitor the disease progression and guide the treatment (7). Furthermore, virtual neurological examination, remote ventilatory monitoring, cognitive evaluation, and psychiatric assessment of the patients could be applied by videoconference technology, however, some limitations need to be investigated (8).

An Italian study evaluated the effect of the COVID-19 pandemic on teaching clinical activities, telemedicine usage, and research activities among neurology residents. Before the pandemic, most of the residents were unfamiliar with telemedicine and face great challenges. However, during the pandemic, about 32% not used the technology for patient care, 35% only used it in emergency cases, and 29% used it for follow-up visits. In addition, only 4% of the participants used telemedicine for all patients. They conclude that there were several challenges faced by the residents such as telemedicine training usage which need to be improved extensively (9).

To fully evaluate a patient, especially in a scientific medical specialty, the assessing physician would definitely require a physical examination in addition to the history. Interestingly, the question of whether virtual examination in neurology can be objective and conclusive was initially assessed in 1990 (10, 11). They made a comparison between a junior neurology resident examining the patient physically and a senior registrar using telemedicine to observe the junior while doing the exam. They concluded that the telemedicine exam is at least as good as the physical exam in different aspects of neurological examination (10–12). However, the limitations of neurology examination are still yet to be fully explored, hence each physician providing teleneurology service must have the skill to determine when the patient needs to be examined in person (2). This study aimed to evaluate the neurology consultants', specialists', and residents' experience and perception of utilizing virtual clinics in neurological assessment in Saudi Arabia.

## 2. Methods

### 2.1. Design, setting, and participants

This cross-sectional study was conducted using a non-probability convenient sampling technique that targeted neurology consultants, specialists, fellows, and residents in Saudi Arabia. An anonymous electronic survey was sent to the neurology practicing physicians through multiple WhatsApp groups of neurologists in Saudi Arabia giving the lack of access to the complete database of contact information of the targeted group of physicians. These groups include neurology residents/fellows and multiple groups of consultants/specialists in all regions. Additionally, all neurology residency training program directors were asked to forward the survey to the residents and colleagues in the center. The survey was sent with multiple reminders from September 2021 until December 2021. The inclusion criteria consisted of neurologists and neurology residents in Saudi Arabia who consented to participate in the study. The exclusion criteria included medical interns and physicians in specialties other than neurology.

### 2.2. Data collection instrument

The questionnaire was developed by two authors based on their clinical experiences. The process started by assessing the confidence level of neurologists and neurology residents in virtual clinics. Afterward, the authors started to develop the parts of the questionnaire composed of 34 items spread across three main sections—demographics, subspeciality, and years of experience, and virtual clinic during the COVID-19 pandemic, with three questions for demographics, two questions for subspeciality and years of experience, and 29 questions for virtual clinic during the pandemic. Questions were designed to evaluate the confidence level and acceptability of neurology virtual clinics. All neurology subspecialties were included in the questionnaire questions starting from new patients in each subspeciality to the follow-up cases. The last part of the questionnaire was added to assess the limitations and possible future improvements that can be applied to virtual clinics or patient encounters. The survey is provided in Supplementary material. Finally, the collected data was organized in Microsoft Excel software to identify any missing data and prepare for analysis.

### 2.3. Ethical considerations

The study was approved by the King Abdullah International Medical Research Center Institutional Review Board. The participants were informed about the purpose of the study and the confidentiality of the research data. Informed consent was obtained from all the participants.

### 2.4. Data analysis

In this study, the IBM statistical package for social sciences statistics (SPSS version 28.0.1.1) and J Macintosh Project pro (JMP version 15.2.0) were used for data analysis. Regarding descriptive categorical data, gender, age, position, years of experience, experience in virtual clinics, and current experience in virtual clinics were all represented by frequencies and percentages. Moreover, descriptive numerical data, such as the scale means, were represented by mean and standard deviation. Regarding inferential statistics, the chi-square test was used for comparison between qualitative variables. The *t*-test and the one-way Analysis of Variance (ANOVA) test were used for analysis between qualitative and quantitative variables. A correlation test was used to identify the level of continuation of the virtual clinic with each neurological subspecialty. The level of significance was set at a *P* < 0.05. The internal consistency was assessed by Cronbach's alpha (α = 0.92).

## 3. Results

The estimated total number of neurology practicing physicians in Saudi Arabia is 848 which includes 341 consultants and 219 specialist/fellows according to the Ministry of Health (MOH) statistical yearbook 2021 (13), and 288 neurology residents in training as per direct communication with Saudi Commission for Health Specialties (SCFHS). Only 108 (12.7%) responded to the survey. Among them, 47 (44%) participants were aged between 30 and 39 years, and 45 (42%) were aged between 20 and 29 years. Approximately 55% of the respondents identify as men. The title/position of the participants in this study was “resident,” “fellow,” “specialist,” and “consultant,” with most of the respondents being residents (62%). The most common subspecialties of the respondents were general neurology and epilepsy with a total number of the two subspecialties 19 (46%). Moreover, the years of neurology experience after residency was categorized as <5 years, 5–10 years, 11–15 years, 15–20 years, or more than 20 years. Seventeen (41%) out of 41 participants had 5–10 years of clinical neurology practice, and most respondents [81 (75%)] had an experience in a neurology virtual clinic. Furthermore, data on the current experience with the neurology virtual clinic revealed 66 (61%) of the respondents only used phones for consultation. Table 1 presents the basic demographic characteristics of the participants.

**Table 1:** Basic characteristics of participants.

**Variable**	***N* = 108**
**Age (*****n*** = **108)**	
20–29	45 (42%)
30–39	47 (44%)
40–49	11 (10%)
50–59	2 (2%)
60–69	3 (3%)
**Gender (*****n*** = **108)**	
Female	48 (44%)
Male	60 (56%)
**Position (*****n*** = **108)**	
Consultant	32 (30%)
Fellow	3 (3%)
Resident	67 (62%)
Specialist	6 (6%)
**Subspeciality for consultants, specialists, and fellows (*****n*** = **41)**	
General neurology	10 (24%)
Epilepsy	9 (22%)
Demyelinating diseases/MS	4 (10%)
Stroke	4 (10%)
Movement disorders	2 (5%)
Neuro-muscular diseases	2 (5%)
Multi-specialty including two or more of the above	10 (24%)
**Years of experience after residency (*****n*** = **41)**	
<5 years	12 (29%)
5–10 years	17 (41%)
11–15 years	6 (15%)
15–20 years	4 (10%)
More than 20 years	2 (5%)
**Past experience with virtual clinic (*****n*** = **108)**	
No	27 (25%)
Yes	81 (75%)
**Current experience with virtual clinic (*****n*** = **108)**	
Only phone	66 (61%)
Video calls	3 (3%)
Both of the mentioned	16 (15%)
None	23 (21%)

Regarding healthcare provided virtually for new patients in each subspecialty, it was found that epilepsy had the highest scale mean of 3.23 [95% confidence interval (CI) = 2.99–3.47], followed by headache, with a scale mean of 3.22 (95% CI = 2.98–3.46) with 64% agreement for each. Care provided for patients with neuromuscular diseases had the lowest scale mean of 1.94 (95% CI = 1.76–2.11), followed by demyelinating diseases/multiple sclerosis (MS), with a mean of 2.17 (95% CI = 1.99–2.36). For follow-ups, epilepsy showed the highest scale mean [mean, 4.44 (95% CI = 4.29–4.59)], followed by headache [mean, 4.41 (95% CI = 4.27–4.57)]. The percentages of the agreement for epilepsy and headache care to be applied virtually for follow-up patients were 88.8% and 88.2%, respectively. On the contrary, the analysis showed the lowest scale mean was for neuromuscular disorders [mean of 3.18 (95% CI = 2.97–3.39)], followed by movement disorders [mean of 3.43 (95% CI = 3.21–3.67)] when applying virtual clinic follow-ups for patients (data summarized in Table 2). Comparison between all subspecialties for the new and follow-up patients revealed a significant difference when comparing each subspecialty means of the virtual clinic for new patients against the corresponding mean for virtual clinic follow-ups (Table 2). All subspecialties included in this analysis showed higher scale means to provide health services for follow-up patients than for new patients in a virtual setting.

**Table 2:** Areas you believe tele neurology can be applied and provide proper care to new and follow-up patients.

**Area**	**New patients**			**Follow-up patients**		***P* value**
	**Scale mean**	**% of agreement**	**Area**	**Scale mean**	**% of agreement**	
General neurology	2.46	49.2	General neurology	4.05	81	0.001
Stroke	2.37	47.4	Stroke	4.17	83.4	0.01
Movement disorders	2.22	44.4	Movement disorders	3.43	68.6	0.001
Dementia	2.76	55.2	Dementia	4.07	81.4	0.001
Demyelinating disease	2.17	43.4	Demyelinating disease	3.61	72.2	0.01
Epilepsy	3.23	64.4	Epilepsy	4.44	88.8	0.001
Headache	3.22	64.4	Headache	4.41	88.2	0.001
Neuromuscular	1.94	38.8	Neuromuscular	3.18	63.3	0.001

**Figure 1:**
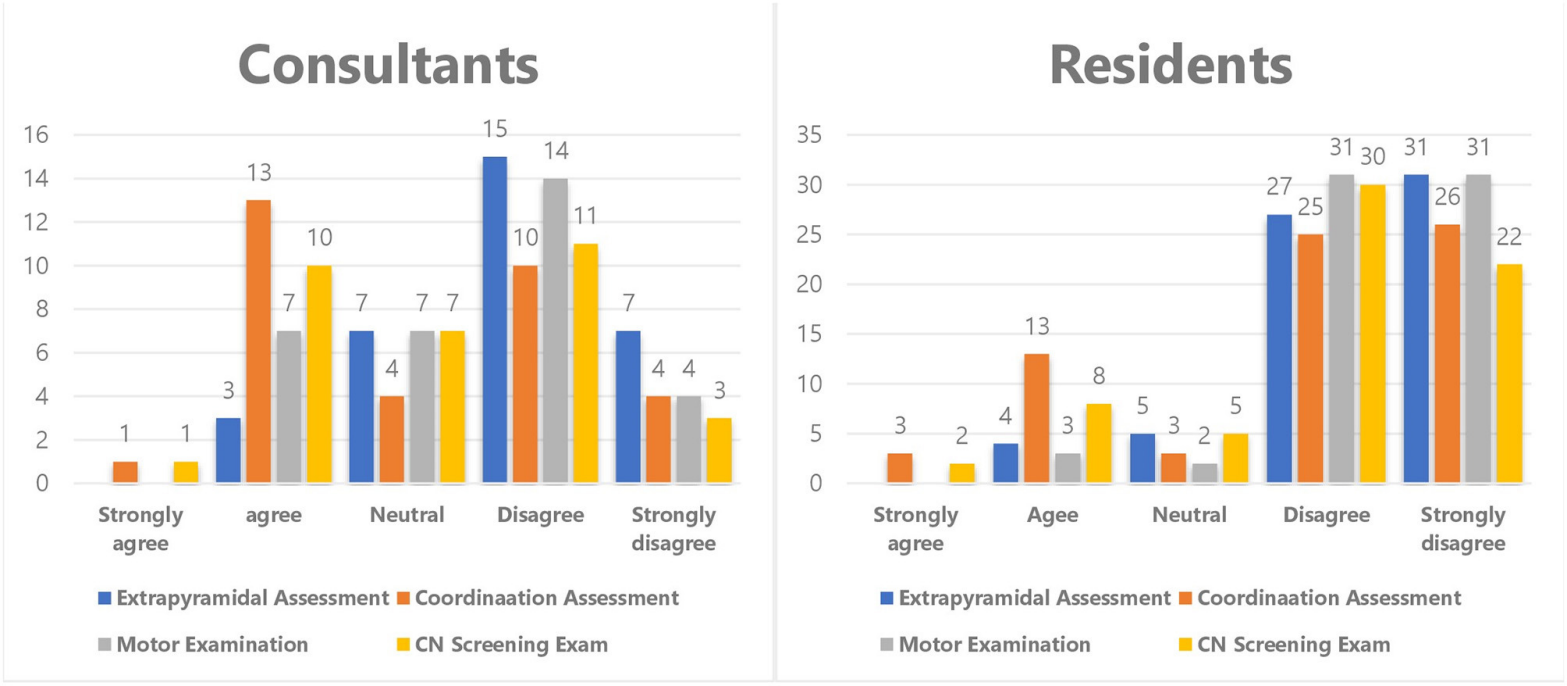
Illustrates the comparison between neurology consultants and residents in performing extrapyramidal assessment, coordination assessment, motor examination, and CN nerve screening. There was statistically significant difference between the consultant and residents in performing these four duties with the consultants being more confident to perform them virtually than the residents.

Statistical analysis revealed that the level of confidence among the neurology physicians for virtual history-taking was the highest, with a scale mean of 4.12 (95% CI = 3.95–4.29), while sensory examination had the lowest scale mean of 1.64 (95% CI = 1.49–1.78) as shown in Table 3. A bivariate analysis for comparison between consultants and neurology residents revealed a significant association between the position and the confidence level in applying cranial nerve exams, motor exams, coordination assessments, and extrapyramidal assessments (*P* < 0.05). It was found that consultants were more confident in performing cranial nerve examination, with a mean of 2.84 (95% CI = 2.47–3.22), which was higher than that of neurology residents [mean of 2.07 (95% CI = 1.81–2.43), *P* < 0.0013]. Additionally, consultants were less doubtful when performing motor examination [mean, 2.53 (95% CI = 2.24–2.82)] than residents, who had a mean of 1.67 (95% CI = 1.46–1.86, *P* < 0.0001). Moreover, consultants could perform the coordination assessment more confidently than residents, with a mean of 2.91 (95% CI = 2.47–3.34) and 2.18 (95% CI = 1.88–2.48), respectively (*P* < 0.0074). Finally, with regard to the extrapyramidal assessment, it was revealed that the consultants were more certain than the residents, with respective means of 2.17 (95% CI = 1.85–2.46) and 1.73 (95% CI = 1.52–1.94) (*P* < 0.0252). Figure 1 demonstrates the distribution of the neurology consultants and residents for the four neurological tasks in which there was a significant difference. However, there was no significant difference observed regarding the relationship between position and history-taking, counseling, mental status exam, sensory examination, and gait assessment.

**Table 3:** Confidence level in performing different parts of neurological assessment virtually.

**Area**	**Scale mean**	**% of agreement**
History taking	4.12	82.4
Counseling services	4.01	80.2
Mental examination	2.86	57.2
Cranial nerves examination	2.29	45.8
Motor examination	1.93	38.6
Sensory examination	1.64	32.8
Extrapyramidal assessment	1.86	37.2
Coordination assessment	2.41	48.2
Gait assessment	2.81	56.2

The bivariate analysis showed no significant relationship between sex, experience of the virtual clinic, and current experience in the virtual clinic with the continuation of the virtual clinic. On the other hand, there was a significant association between position and years of experience after residency and the continuation of virtual clinics. Fellows and consultants showed a high desirability to continue the virtual clinics, with means of 4 (95% CI = 2.74–5.25) and 3.91 (95% CI = 3.52–4.29), respectively. In addition, in regard to years of experience after residency, those who had 11–15 years' experience exhibited a higher mean to continue the clinics virtually [4.17 (95% CI = 3.74–4.59)]. Table 4 summarizes the results of different basic variables in comparison to future virtual clinic continuation.

**Table 4:** Virtual clinic continuation by basic characteristics of participants.

**Variable**	***N* = 108**	***P* value**
**Mean**		
**Gender**		
Female	3.43	0.607
Male	3.55	
**Position**		
Consultant	3.91	0.03
Fellow	4.00	
Resident	3.25	
Specialist	3.83	
**Years of experience after residency**		
<5 years	4.07	0.021
5–10 years	4.00	
11–15 years	4.17	
15–20 years	4.00	
More than 20 years	1.50	
**Past experience with virtual clinic**		
No	3.48	0.925
Yes	3.51	
**Current experience with virtual clinic**		
Only phone	3.38	0.355
Video calls	3.67	
Both	3.93	
None	3.52	

The correlation test between the virtual clinic continuation and the subspecialties for the new and follow-up patients showed a significant correlation for most clinics. However, no significant relationship was found between the continuation level with new patients in demyelinating diseases and neuromuscular clinics (*r* = 0.151 and 0.160, respectively). In contrast to demyelinating diseases clinics for new patients, a strong correlation was noted in the level of continuation for the follow-up patients (*r* = 0.310), while the neuromuscular clinics for follow-up patients showed a weak correlation in the level of continuation (*r* = 0.170) (details for the correlation test are illustrated in Table 5).

**Table 5:** Correlation between virtual clinic continuation and subspecialties.

**Area**	**r (new)**	***P* value**	**r (follow-up)**	***P* value**
General neurology	0.217	0.023	0.406	0.001
Stroke	0.355	0.001	0.428	0.001
Movement disorders	0.194	0.043	0.229	0.001
Dementia	0.332	0.001	0.378	0.016
Demyelinating disease	0.151	0.119	0.310	0.001
Epilepsy	0.349	0.001	0.432	0.001
Headache	0.205	0.032	0.449	0.001
Neuromuscular	0.160	0.098	0.170	0.078

Regarding the limitations that applied to the virtual clinics in neurology, neurology practitioners addressed that patient experiences and physician acceptance were the two main limitations to occur in virtual clinics [60 (55.6%) for each]. Other addressed limitations observed in infrastructure, include the internet [58 (53.7%)], policies and regulations [38 (35%)], and budget [20 (18.5%)].

## 4. Discussion

The pandemic has forced the healthcare sector to modify its outpatient care system in order to adapt to the newly encountered challenges. Telemedicine, which was one of the available options, was used to assess patients' health and was implemented instantly in practice without additional delay owing to preparations. This immediate shift demonstrated that such radical changes to overcome any abrupt challenges, if perceived as crucial, are feasible. However, the lack of assessment of these rapid modifications in healthcare practice is still an issue; regardless, it is necessary to follow the patients' and physicians' experiences and acceptance, as well as address the factors that might affect quality-of-care.

This investigation provided an opportunity to evaluate neurology-practicing physicians' perceptions regarding telemedicine during the pandemic in Saudi Arabia. The outcomes of this study revealed that the acceptability and confidence in the neurology virtual clinics favored history-taking over physical examinations, and they were more suitable for follow-up appointments rather than newly referred patients. Additionally, it was found that virtual clinics were more appropriate for patients with epilepsy and headache than those with other subspecialty disorders, according to the neurologists' perspective.

Epilepsy and headache were the two most commonly seen neurological conditions, both in new and follow-up patients in virtual outpatient clinics. Regarding primary headache disorders, the results of the current study confirm the findings from previous studies implementing that it may be well suited for teleconsultation. A randomized trial evaluating telemedicine services for migraine management (14) found that migraine-related disability, number of days with headache, and severity showed no significant difference between patients receiving management through telemedicine and those receiving on-site consultations. Furthermore, the trial used a five-point Linkert scale, which showed significant differences in the scale mean for the convenience of telemedicine visits (4.7) compared with that of on-site consultations (3.5). It has been denoted that telemedicine in epilepsy is also satisfactory, suggesting that the virtual clinical assessment is as effective as standard care with regard to control and drug adherence. In line with the current findings, a study by Rasmusson et al. (15) showed that care outcomes (number of episodes, visits to the emergency room, hospitalization, and medication compliance) between patients with epilepsy who received care through telecommunication in comparison to in-person clinics found that no significant difference in outcome between the two groups. These findings support the continuation of headache and epilepsy clinics virtually, as they are mainly diagnosed by history-taking with minimal physical examination (16). Moreover, the results of this study showed that dementia clinics are the third preferred clinic to be conducted remotely after headache and epilepsy for new patients, however, it is ranked the fourth after stroke in follow-up settings. Generally, the scale means of dementia for the assessment of new and follow-up cases were high. This could be demonstrated by developing many diagnostic tools to assess patients with dementia or behavioral conditions remotely such as the modified Montreal Cognitive Assessment (MoCA) (17). As well, as an Australian study aimed to develop a telemedicine protocol to provide healthcare services for Alzheimer's disease. It was found that performing the Mini-Mental Status Exam (MMSE) and the Geriatric Depression Scale virtually, which are used also for patients with dementia and cognitive impairment, were effective as face-to-face clinics (18).

On the contrary, applying and continuing teleneurology after the pandemic for the care of neuromuscular disorders, movement disorders, and demyelinating diseases/MS had the lowest percentages of agreement from neurology physicians, as it was considered insufficient in providing proper care for patients. Similarly, findings described by Kristoffersen et al. (16) note that 94% of the physicians do not prefer encountering new patients with MS virtually, while only half were satisfied with the experience for follow-up cases. In addition, they found that care provided to patients with movement disorders was not desired among 57% of the participating physicians, and 96% refused to assess new referrals. Another study by McKenna et al. found that 66.7% of patients with neuromuscular disorders prefer to attend the on-site clinic since their conditions require physical examination (19). However, the Vasta et al. study, which is a survey-based study that aimed to investigate the satisfaction of patients with amyotrophic lateral sclerosis (ALS) and their caregivers with telemedicine during the pandemic, showed that most of the participants were satisfied with the telemedicine services. However, this conclusion was based on follow-up consultation tasks such as asking about the next clinic appointments and consultations regarding the management plan (20). This is expected as these conditions require a more comprehensive physical exam, which cannot be properly conducted in virtual clinics (16).

Generally, our findings indicate that virtual clinics are considered to be more satisfactory for follow-up patients than for new referrals, which is consistent with the results described by Kristoffersen et al., seeing the unsuitability of teleconsultation for poorly defined and unpredictable cases (16). This was noted in both general and across all neurology subspecialties, likely due to the possibility of misdiagnosis and the resulting incorrect management plan, or alternatively, due to other disadvantages, such as difficulty in establishing trust virtually to build the physician-patient relationship, privacy issues, or missing critical information provided non-verbally.

The level of confidence among neurologists and neurology residents for virtual history-taking was higher than for physical examination. Virtual physical exams might pose challenges, owing to multiple factors, including the clinical skill needed to perform the exam, patients' knowledge and cooperation, and the means used for communication. The most used teleconsultation means in virtual clinics, according to this study, were telephones. This might suggest that phones are more easily accessible and convenient for both physicians and patients, but also indicates limitations for the physician's ability to evaluate patients' health properly in regard to detailed physical examination. However, more confidence was still observed among consultants in performing cranial nerve examinations virtually; furthermore, consultants were more confident in performing motor examinations than neurology residents. These findings are in line with those outlined by Kristoffersen et al., who noted that the consultants can handle the physical assessment more appropriately than residents, albeit the assumption that younger residents would be more familiar with updates in technology in general (16). This might be due to experience, as it was indicated that consultants have a higher desire to continue providing health services through virtual clinics after the resolution of COVID-19 than residents. It is suggested that telemedicine training sessions be embedded in the neurology residency training program curriculum to enhance the residents' competency in treating the patients virtually (9).

According to the present results, patients' experiences and physicians' acceptance were the main barriers to the virtual neurology clinics implementation. In a study by Kumar et al., 52% of the 1,388 participating patients preferred to return to face-to-face consultations once the COVID-19 pandemic would have ended (21). Another study found that 65% of neurology physicians prefer in-person counseling over telecommunication clinics (16). For proper neurological examination, techniques require clear instructions from the clinician and high levels of cooperation from the patient and their families. When observing tasks being performed by the patient or communicating with the family, solely virtual assessments may mislead physicians into omitting important aspects potentially affecting the diagnosis. Additionally, some patients, in particular older adults, may have difficulties using smartphones, which can impact the completeness of the virtual visit (3). There is a need for more in-depth future investigations on patients' outcomes, perceptions, and experiences of telemedicine in Saudi Arabia.

The main limitation of this study was the sample size. Respondents to the survey might not entirely be representative of the population, considering that most neurologists lack time to fill out the survey due to long working hours in the hospitals, teaching duties, or not experiencing virtual clinics. The exact response rate could not be calculated properly due to the lack of complete access to the database. We note no reason to suspect response bias; however, there was a possibility of procedural bias, owing to the limited time for most of the respondents. Finally, recall bias might have occurred during questionnaire filling.

## 5. Conclusion

Virtual clinics were rapidly implemented during the COVID-19 pandemic in Saudi Arabia to follow the social distancing and precautionary measures implemented to control the spread of the infection. In neurological clinical practice, most neurology-practicing physicians showed more confidence in performing history-taking tasks virtually than in physical examination. However, the consultants were more confident in performing the neurological examination than neurology residents. Furthermore, headaches and epilepsy were considered to be more suitable conditions to be handled through telecommunication than other subspecialties. Additionally, teleconsultations for follow-up patients were seen as more satisfactory than their application for newly referred patients. This study suggested that virtual clinics can be utilized even after the COVID-19 situation, especially with follow-up patients in some subspecialties such as epilepsy and headache. Also, further training in telemedicine is required especially for neurology residents as they will shape the future of neurological consultation. Further studies with larger sample sizes are needed to gain new insights into the level of confidence in performing different duties with regard to neurology virtual clinics.

## Data availability statement

The original contributions presented in the study are included in the article/Supplementary material, further inquiries can be directed to the corresponding author.

## Ethics statement

The studies involving human participants were reviewed and approved by King Abdullah International Medical Research Center Institutional Review Board. The patients/participants provided their written informed consent to participate in this study.

## Author contributions

All authors listed have made a substantial, direct, and intellectual contribution to the work and approved it for publication.
