# Supplementary material for: Experience and perception of utilizing virtual clinic in neurological assessment in Saudi Arabia
Source: Front Neurol. 2023 Feb 17;14:1111254. doi: 10.3389/fneur.2023.1111254 (PMC9983690; doi:10.3389/fneur.2023.1111254)
Supplement: Supplementary file 1 [file Data_Sheet_1.PDF]

NRJ21J/181/07

Experience and Perception of Utilizing Virtual Clinic in Neurological Assessment in Saudi Arabia **Questionnaire**

King Abdullah International Medical Research Center
KAIMRC
APPROVED
IRB
Institutional Review Board

<u>Demographics</u>

1. Age
2. Gender: Male ( ) / Female ( )
3. Title or position:
    a. Resident: R1 ( )/ R2 ( )/ R3 ( )/ R4 ( )/ R5 ( )
    b. Fellow ( )
    c. Specialist/ Staff ( )
    d. Consultant
        i. Subspecialty/s (multiple options can be chosen)
            1. Stroke
            2. Movement disorders
            3. Dementia
            4. Demyelinating diseases/ MS
            5. Epilepsy
            6. Headache
            7. Neuro-muscular
            8. Others:
            9. None

        ii. Years of experience (After residency)
            1. Less than 5 years
            2. 5- 10 years
            3. 11- 15 years
            4. 15 – 20 years
            5. More than 20 years

During COVID19 pandemic:

- Have you worked in a virtual clinic, or used teleneurology to provide care for your patients?
    a. Yes ( )
    b. No ( )
- Current experience
    a. Only phone
    b. Video calls
    c. None
    d. Others:

- What are the areas you believe teleneurology can be applied and provide proper care to **NEW** patients
    a. Outpatient- General Neurology:
        1. Strongly disagree
        2. Disagree
        3. Neutral
        4. Agree
        5. Strongly Agree

    b. Stroke:
        1. Strongly disagree
        2. Disagree
        3. Neutral
        4. Agree
        5. Strongly Agree

    c. Movement disorders:
        1. Strongly disagree
        2. Disagree
        3. Neutral
        4. Agree
        5. Strongly Agree
    d. Dementia:
        1. Strongly disagree
        2. Disagree
        3. Neutral
        4. Agree
        5. Strongly Agree

    e. Demyelinating disease/ MS:
        1. Strongly disagree
        2. Disagree
        3. Neutral
        4. Agree
        5. Strongly Agree
    f. Epilepsy:
        1. Strongly disagree
        2. Disagree
        3. Neutral
        4. Agree
        5. Strongly Agree
    g. Headache:
        1. Strongly disagree
        2. Disagree
        3. Neutral
        4. Agree
        5. Strongly Agree

h. Neuromuscular:
    1. Strongly disagree
    2. Disagree
    3. Neutral
    4. Agree
    5. Strongly Agree

- What are the areas you believe teleneurology can be applied and provide proper care to **FOLLOW-UP** patients
    a. Outpatient- General Neurology:
        1. Strongly disagree
        2. Disagree
        3. Neutral
        4. Agree
        5. Strongly Agree

    b. Stroke:
        1. Strongly disagree
        2. Disagree
        3. Neutral
        4. Agree
        5. Strongly Agree
    c. Movement disorders:
        1. Strongly disagree
        2. Disagree
        3. Neutral
        4. Agree
        5. Strongly Agree
    d. Dementia:
        1. Strongly disagree
        2. Disagree
        3. Neutral
        4. Agree
        5. Strongly Agree

    e. Demyelinating disease/ MS:
        1. Strongly disagree
        2. Disagree
        3. Neutral
        4. Agree
        5. Strongly Agree
    f. Epilepsy:
        1. Strongly disagree
        2. Disagree
        3. Neutral
        4. Agree
        5. Strongly Agree

g. Headache:
    1. Strongly disagree
    2. Disagree
    3. Neutral
    4. Agree
    5. Strongly Agree
h. Neuromuscular:
    1. Strongly disagree
    2. Disagree
    3. Neutral
    4. Agree
    5. Strongly Agree

Rate your confidence level in performing the following through <u>virtual clinic</u>:

- Taking History from neurology patients

1. Strongly disagree
2. Disagree
3. Neutral
4. Agree
5. Strongly Agree

- Counseling for your patients

1. Strongly disagree
2. Disagree
3. Neutral
4. Agree
5. Strongly Agree

➢ In an online patient encounter **<u>I feel more confident in performing</u>**

- Mental status examination

1- Strongly disagree
2- Disagree
3- Neutral
4- Agree
5- Strongly Agree

- Cranial nerves screening exam

1. Strongly disagree
2. Disagree
3. Neutral
4. Agree
5. Strongly Agree

- Motor Examination

1. Strongly disagree
2. Disagree
3. Neutral
4. Agree
5. Strongly Agree

- Sensation

1. Strongly disagree
2. Disagree
3. Neutral
4. Agree
5. Strongly Agree

- Extrapyramidal

1. Strongly disagree
2. Disagree
3. Neutral
4. Agree
5. Strongly Agree

- Coordination

1. Strongly disagree
2. Disagree
3. Neutral
4. Agree
5. Strongly Agree

- Gait

1. Strongly disagree
2. Disagree
3. Neutral
4. Agree
5. Strongly Agree

- Do you think **virtual clinics in neurology** should be continued after resolution of COVID-19 Future expectations:

1. Strongly disagree
2. Disagree
3. Neutral
4. Agree
5. Strongly Agree

King Abdullah International Medical Research Center
KAIMRC
APPROVED
IRB
Institutional Review Board

- Main limitation/s to applying virtual clinics in neurology:
    a. Budget
    b. Policies and regulations
    c. Infrastructure including internet
    d. Patient acceptance
    e. Physician experience and acceptation
    f. Others:
